# The Effect of Diagnostic Delays on the Drop-Out Rate and the Total Delay to Diagnosis of Tuberculosis

**DOI:** 10.1371/journal.pone.0001933

**Published:** 2008-04-09

**Authors:** Stephen J. Millen, Pieter W. Uys, John Hargrove, Paul D. van Helden, Brian G. Williams

**Affiliations:** 1 DST/NRF Centre of Excellence for Epidemiological Modelling and Analysis (SACEMA), Stellenbosch University, Western Cape, Republic of South Africa; 2 Division of Molecular Biology and Human Genetics, MRC Centre for Molecular and Cellular Biology, DST/NRF Centre of Excellence for Biomedical TB Research, Faculty of Health Sciences, Stellenbosch University, Western Cape, Republic of South Africa; 3 Stop TB, World Health Organization, Geneva, Switzerland; Instituto de Pesquisa Clinica Evandro Chagas, FIOCRUZ, Brazil

## Abstract

**Background:**

Numerous patient and healthcare system-related delays contribute to the overall delay experienced by patients from onset of TB symptoms to diagnosis and treatment. Such delays are critical as infected individuals remain untreated in the community, providing more opportunities for transmission of the disease and adversely affecting the epidemic.

**Methodology/Principal Findings:**

We present an analysis of the factors that contribute to the overall delay in TB diagnosis and treatment, in a resource-poor setting. Impact on the distribution of diagnostic delay times was assessed for various factors, the sensitivity of the diagnostic method being found to be the most significant. A linear relationship was found between the sensitivity of the test and the predicted mean delay time, with an increase in test sensitivity resulting in a reduced mean delay time and a reduction in the drop-out rate.

**Conclusions/Significance:**

The results show that in a developing country a number of delay factors, particularly the low sensitivity of the initial sputum smear microscopy test, potentially increase total diagnostic delay times experienced by TB patients significantly. The results reinforce the urgent need for novel diagnostic methods, both for smear positive and negative TB, that are highly sensitive, accessible and point of care, in order to reduce mean delay times.

## Introduction

Tuberculosis (TB) is currently the world's leading cause of death from a single infectious condition. Despite widespread introduction of the Directly Observed Therapy Short-course (DOTS) program, heralded as “one of the major public health success stories of the past decade”, incidence of the disease continues to rise in many sub-Saharan African countries, exacerbated by high levels of HIV. In 2005, the estimated TB case load in South Africa alone was over 470, 000.[1]

Case detection, specifically excessive delays in correct TB diagnosis and treatment remains a weakness of the control strategy and has been the focus of much research [2]–[4]. Such delays result in greater opportunity for patients to infect susceptible individuals and may contribute significantly to the high incidence levels evident in many developing countries [1], [5]–[7]. In order to control the TB epidemic it is therefore vitally important to identify and reduce these delays. This implies that it is necessary to fully understand the causes of these delays and to estimate their magnitudes so as to enable the planning of interventions that yield the maximum benefit.

Before correct diagnosis is achieved and treatment started there are a large number of delay factors that contribute to an overall delay time. There are inherent delays in the current diagnostic process such as time taken to collect, transport and examine sputum samples for sputum smear microscopy (SSM). However a TB case may also experience any one of a large number of sequences of events or circumstances that contribute to the total delay and are subject to a certain amount of random variation. Such events or circumstances are largely associated with the care seeking behaviour of patients. [8]

A simulation model was developed to enable an investigation to be carried out into the relative importance of each of the potential delays. We use the model to calculate the total time, from onset of symptoms until diagnosis, based on probabilities for the transition from one event to another and their associated delays. The simulation was repeated a number of times to generate a delay time distribution and the sensitivity of this to each parameter, be it a transition probability or an associated delay, could then be determined.

## Methods

A decision tree simulation model was used to analyse the delays that occur between the onset of TB symptoms through to diagnosis and treatment. All individuals in the model are assumed to be positive for TB and only pulmonary TB cases are considered, since these have the potential to infect others. We describe below, the various events represented in the model that can contribute to the total delay before reaching a definite diagnosis and the start of treatment.

A prospective patient enters the model after showing symptoms of TB, most commonly a cough, of three weeks duration. The individual then has the option of one of the following:

- Seek no help at all.
- Visit a health care provider.

Those in the former category are assumed to remain infectious in the community until such a time that the patient self-cures or dies. Although such individuals are likely to infect others and as a result lead to a greater number of TB cases to be managed by the healthcare system, this phenomenon is not included in this simulation. Infected individuals that seek a healthcare provider may either:

- Visit a health care provider who has no facility for testing TB.
- Visit a health care provider who can test for TB.

The former includes traditional healers, homeopaths or any form of therapy/treatment which is not centred on standard clinical tests and treatment for TB. Such a healthcare encounter is assumed to have negligible effect on the patient's condition. Having sought a non-TB specific health provider the individual may continue with such therapy, drop out and seek no further care or proceed to seek standard TB-specific healthcare.

In the latter case the infected individual seeks formal TB care. No distinction is made between a dedicated TB clinic and a hospital, be it public or private, but considers only those that practice according to the NTCP (national tuberculosis control programme). Upon visiting the TB healthcare provider the patient may experience any one of the following:

- S/he may be treated for the cough, but not for TB, and discharged.
- A CXR may be taken.
- A sputum sample may be taken for diagnostic tests, and further samples requested.

In the first event the patient may drop out or may, given that symptoms persist, make further visits to the TB-test facility. For simplicity, we exclude the possibility that, having been to a TB-test facility, the patient then goes back to an alternative health care provider.

If a CXR is carried out and is judged to be normal, a negative diagnosis is given and the patient is discharged. If the CXR is abnormal, together with symptoms indicating TB, a positive diagnosis may be made and treatment begun. However the following delays may be incurred:

- The delay in taking the CXR.
- The delay in producing the results.
- The delay in the patient collecting the results.
- The delay in the start of treatment, given a positive diagnosis.

In the event that sputum samples are requested, numerous delays can and do arise:

- The delay until the first sputum sample is taken. This is generally taken on the spot but may not be possible if the patient finds it difficult to expectorate.
- The delay in producing further samples. This should be completed the following day; however this is not always achieved, particularly with patients living in poverty, whose circumstances may make it difficult to access the healthcare provider.
- Delay in the results being received. This includes the delay in transporting the samples to the laboratory, then being examined and the results recorded. Combined this usually takes 12–48 hours. This is another opportunity for the patient to drop out.
- The delay in the patient returning to collect the result, once all of the sputum samples have been provided and the tests completed.
- The delay in the start of treatment, in the event that the patient tests positive.

Despite a patient being positive for TB and receiving the correct tests, due to the low sensitivity of SSM a negative diagnosis may still be given. The simulation allowed us to vary the sensitivity of a diagnostic test over a wide range in order to investigate the theoretical limits to the effect that improved diagnostic techniques could have on treatment delay. It is assumed in the model that TB treatment is not started if the sputum result is negative. In the event of an incorrect diagnosis being given the infected patient may drop out or continue with further testing. A specific algorithm is followed for smear negative patients suspected of having TB. Firstly a chest X-ray is carried out which leads to one of the following:

- The X-ray is normal, indicating a low suspicion of TB. The patient receives a negative diagnosis but may return to the clinic in the future.
- The X-ray is abnormal, which indicates a high suspicion of TB. The patient may be treated presumptively based on the CXR or go on for further testing to confirm the diagnosis.

Additional testing for patients suspected of having TB requires further sputum samples. SSM and culture testing are initiated at this stage; however the latter requires a considerably longer period before results are available. Therefore, in the event of a positive SSM result i.e. smear positive, a diagnosis is made before the culture results are processed, otherwise the patient must wait for up to 8 weeks for a definitive diagnosis. This stage in the diagnostic process represents a very significant proportion of the overall delay time and explains why smear negative patients incur such an excessive delay between the onset of symptoms and diagnosis and treatment and patients may drop out during this time.

The model assumes constant transition probabilities regardless of how many visits a patient has made. In reality this is unlikely to be the case, as in each successive visit there will be changes in the probabilities that patients drop out, of different tests being applied and of which action will be taken given a particular outcome for each test. Thus, for example, the probability that a health care provider decides to treat TB syndromically, even in the face of negative sputum results, will presumably increase with each test. We also assume a constant specific probability each day that a patient makes a visit to the TB test facility. This is equivalent to assuming that the waiting times are exponentially distributed. It is also implied that the mean waiting time between events is equal to the inverse of the daily probability that the event occurs. It is unlikely that the distribution is in fact exponential. One might assume that the longer the patient has had the symptoms the sicker s/he is likely to be and will thus be more likely to visit a health provider on a particular day. In mathematical terms, the hazard is not a constant but increases with time since onset of symptoms. Without definite data regarding the actual distribution the constant probability approach will have to be used. Probabilities for other events are fixed and independent of time.

It will be noted that the results of the two routinely collected sputum specimens are not independent, but rather are highly correlated and this consideration is incorporated in the simulation model design. Thus if the first specimen yields a positive result, i.e. if the specimen exceeds the threshold for the test, then it is likely that the following specimen will also exceed this threshold and be positive. The converse applies when the first specimen yields a negative result. In other words, the probability of a test giving a positive result is not only dependent on the sensitivity of the test but depends also on the quantity of bacteria present in the sputum and hence on the condition of the specific patient.

## Results

**Figure 1:**
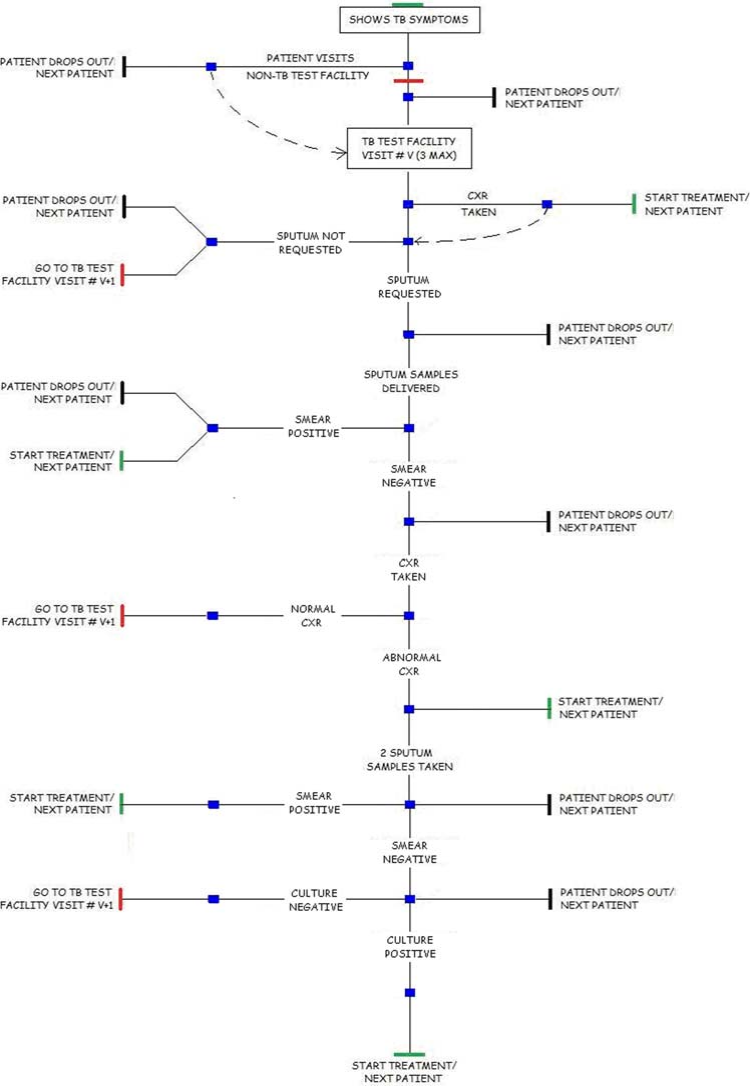
Schematic of the model.

The purpose of this investigation is to quantify the effect of various diagnostic delays on the overall delay experienced by TB patients before treatment is started. To this end the flowchart shown in figure 1 was implemented in an Excel spreadsheet using macros. The total delay experienced by a virtual patient can be evaluated and is dependent on the probabilities and associated delay times at each stage. By repeating the simulation calculation many times a distribution for total delay times can be generated. The process of creating distributions was repeated for various parameter settings and the mean time for each distribution was calculated.

**Figure 2:**
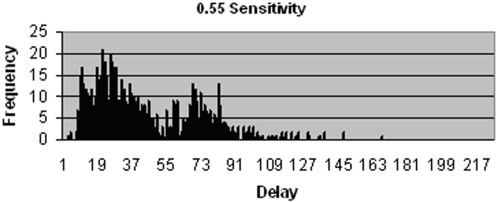
Distribution of delays since onset of symptoms to start of treatment for patients that do not drop out, using a SSM test sensitivity of 0.55.

By running the model with input parameters set to reflect the current diagnostic protocol in South Africa (see Tables 1 and 2), a distribution similar to that seen in figure 2 is produced. This is based on two smear samples being used in the initial SSM test, producing a sensitivity of 0.55. It should be emphasised that estimates based on reports from clinics, in the Western Cape, RSA are used for the input data and that actual documented data needs to be gathered. However the analysis is not critically contingent on precise data values.

**Table 1:** Standard ZN Microscopy Using Two Smears (Pre-test and smear positive parameters)

Parameters	Code Name	Values	Reference
Total number of patients	NTOT	1000	-
P(ith patient has TB)	PTB	1	-
Sensitivity of Initial Diagnostic Procedure	PPV	0.55	36
Maximum number of days+ve patient survives untreated	SURV	1460	VAR
P(patient first visits non-clinic health provider)	NCHP	0.5	VAR
Delay consequent on visit to non-TB test centre	DNCHP	10	VAR
P(makes 1st visit to clinic on any given day)	PHC1	0.05	VAR
P(makes 2nd visit to clinic on any given day)	PHC2	0.05	VAR
P(makes 3rd visit to clinic on any given day)	PHC3	0.05	VAR
P(drops out between visits 0 & 1 I NO sputum taken in visit 0)	PD10	0.01	VAR
P(drops out between visits 1 & 2 I NO sputum taken in visit1)	PD20	0.01	VAR
P(drops out between visits 2 & 3 I NO sputum taken in visit2)	PD30	0.01	VAR
P(drops out before going to clinic for visit 1)	PD11	0.01	VAR
P(drops out between visits 1 & 2 I sputum taken in visit1)	PD21	0.01	VAR
P(drops out between visits 2 & 3 I sputum taken in visit2)	PD31	0.01	VAR
P(clinic asks for CXR at visit 1)	PCXR1	0.10	VAR
P(clinic asks for CXR at visit 2)	PCXR2	0.10	VAR
P(clinic asks for CXR at visit 3)	PCXR3	0.10	VAR
P(clinic treats on basis of CXR at visit 1)	SENCXR1	0.50	VAR
P(clinic treats on basis of CXR at visit 2)	SENCXR2	0.50	VAR
P(clinic treats on basis of CXR at visit 3)	SENCXR3	0.50	VAR
Delay in treating after deciding to treat given CXR result)	DELCXR	2	VAR
P(clinic asks for sputum sample at visit 1)	PS1	0.95	VAR
P(clinic asks for sputum sample at visit 2)	PS2	0.95	VAR
P(clinic asks for sputum sample at visit 3)	PS3	0.95	VAR
P(drops out during visit 1, BEFORE giving sputum)	PD12	0.01	VAR
P(drops out during visit 2, BEFORE giving sputum)	PD22	0.01	VAR
P(drops out during visit 3, BEFORE giving sputum)	PD32	0.01	VAR
P(drops out during visit 1, BEFORE collecting results)	PD13	0.01	VAR
P(drops out during visit 2, BEFORE collecting results)	PD23	0.01	VAR
P(drops out during visit 3, BEFORE collecting results)	PD33	0.01	VAR
Delay in delivering sputum samples at visit 1	DEL11	1	VAR
Delay in delivering sputum samples at visit 2	DEL21	1	VAR
Delay in delivering sputum samples at visit 3	DEL31	1	VAR
Delay in lab producing results after visit 1	DEL12	2	VAR
Delay in lab producing results after visit 2	DEL22	2	VAR
Delay in lab producing results after visit 3	DEL32	2	VAR
Delay in patient collecting results after visit 1	DEL13	2	VAR
Delay in patient collecting results after visit 2	DEL23	2	VAR
Delay in patient collecting results after visit 3	DEL33	2	VAR
Delay in start of treatment after+ve diagnosis	DELTR	2	22, VAR

**Notes**: VAR = Variable/data not available. P(Assertion 1 | Assertion 2) denotes the probability of Assertion 1 being true given the truth of Assertion 2.

**Table 2:** Standard ZN Microscopy Using Two Smears (Smear negative algorithm parameters)

CXR test (smear negative patients)	CXR2	0.72	36
Microscopy test (smear negative patients)	PPV2	0.08	36
Culture test (smear negative patients)	PPV3	1.00	36
P(smear neg. patient drops out before CXR is carried out)	PDN	0.01	VAR
P(smear neg. patient drops out before collecting CXR results)	PDRN	0.01	VAR
P(smear neg. patient drops out before collecting micr. results)	PDRNM	0.01	VAR
P(smear neg. patient drops out before collecting culture results)	PDRNC	0.05	VAR
P(decide to treat presumptively based on abnormal CXR)	TRCXR	0.10	VAR
Delay in lab producing smear neg. patient's CXR results	DELN	0	VAR
Delay in lab producing smear neg. patient's micr. results)	DELM	2	VAR
Delay in lab producing smear neg. patient's culture results	DELC	42	34
Delay in smear neg. patient collecting CXR results	DELPN	0	VAR
Delay in smear neg. patient collecting micr. results	DELPM	1	VAR
Delay in smear neg. patient collecting culture results	DELPC	5	VAR
Cost per AFB smear test (US$) (single specimen test)	CAFB	4.01	25
Cost per Chest X-ray (US$)	CCXR	6.60	25
Cost per LJ Culture/differential (US$)	CLJC	19.04	*
Cost per Clinic Visit (US$)	CCLV	3.90	25

**Notes**: VAR = Variable/data not available ^*^ NHLS Laboratory Costs (2004) P(Assertion 1 | Assertion 2) denotes the probability of Assertion 1 being true given the truth of Assertion 2.

Two groupings can clearly be seen within the distribution shown in figure 2. The left hand cluster represents patients that are diagnosed using the initial SSM test, whereas the cluster to the right represents smear negative TB cases, largely diagnosed by the culture test. The latter group incur significantly longer delays due to the long period of time required to culture Mycobacterium.

**Figure 3:**
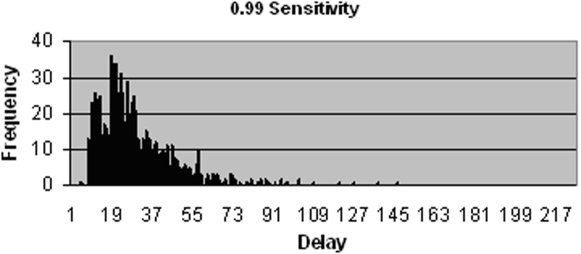
Distribution of delays since onset of symptoms to start of treatment for patients that do not drop out, using a SSM test sensitivity of 0.99.

**Figure 4:**
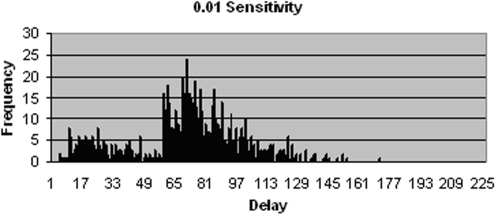
Distribution of delays since onset of symptoms to start of treatment for patients that do not drop out, using a SSM test sensitivity of 0.01.

The TB patients that fail to be identified by the initial SSM test and who thus experience much longer delays form part of the cluster to the right in figure 2. The relative size of this cluster is dependent on the sensitivity of the test. When initial SSM test sensitivity is increased to 0.99, as shown in figure 3, a majority of patients receive a diagnosis based on this test and thus fall within the left hand cluster. The distribution reveals an offset to the overall delay time, with the minimum diagnostic delay at approximately 10 days. This is due to the inherent delays in the diagnostic process, such as that associated with patients producing both samples, technicians examining sputum smears and producing the results, and the start of treatment following a positive diagnosis. This minimum delay is associated with just one of the many possible sequences of events that precede diagnosis. Delay between onset of symptoms and start of treatment is exponentially distributed due to transition probabilities that are independent of time, such as the constant probability that a patient will make a visit to the clinic on a given day. The interruption that can be seen in the pattern at approximately day 15 is due to a group of patients, positioned further to the left, who are diagnosed by the SSM test on their first visit and a group to the right, starting on approximately day 20, who are diagnosed also by the SSM test, but after making a second or sometimes third visit to the clinic. Conversely when initial SSM test sensitivity is decreased significantly, as shown in figure 4, the proportion of TB patients who are smear negative increases, meaning they incur additional delays as they must wait for further test results. Due to the low sensitivity of the additional SSM test, a majority of smear negative patients must wait for culture results, which takes approximately 6 weeks on average to produce.

**Figure 5:**
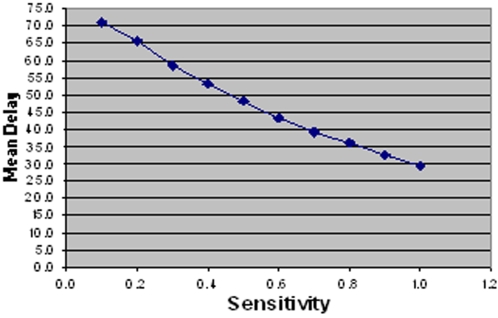
Linear relationship between initial SSM test sensitivity and mean number of days delayed between onset of symptoms and start of treatment for those that did not drop out.

From these histograms it is clear that test sensitivity has a significant effect on delays, specifically by influencing the proportion of patients that receive a diagnosis from the initial SSM test. However in order to quantify this effect more precisely, mean delay was calculated from the distribution. By varying the sensitivity of SSM (initial test for all patients) a linear relationship with delays is produced, as shown in figure 5. Mean delay is reduced by approximately 3 days for every 0.1 increase in test sensitivity when this approaches 1.0 and 5 days when sensitivity is very low. The mean delay ranges from 29.4 days when test sensitivity is 1.0 to 70.9 days with a sensitivity of 0.1.

**Figure 6:**
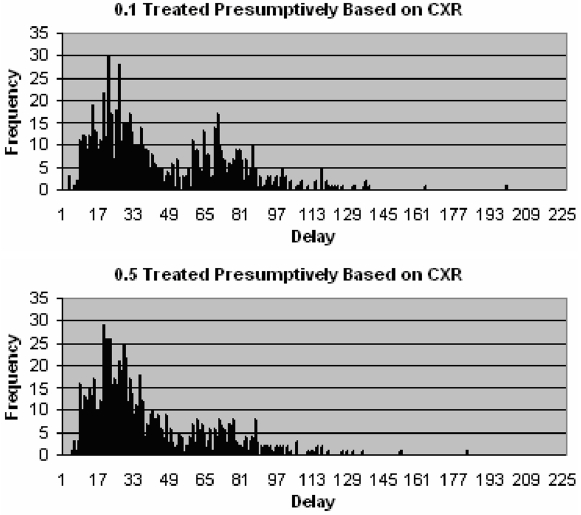
Distribution of delays since onset of symptoms to start of treatment for patients that do not drop out, when 0.1 (above) and 0.5 (below) smear negative patients with an abnormal CXR are treated presumptively.

It is apparent that the mean length of delays experienced by patients is greatly affected by the proportion of smear negative patients. In order to further investigate the effect that the smear negative algorithm has on delays, we examined other parameters. By altering the proportion of smear negative patients that are treated presumptively based on CXR results, the number of patients that have to wait for culture results also changes. Due to the aforementioned long delay associated with culture this has a significant effect. The frequency of longer delays is reduced when 50% of smear negative cases are treated presumptively when compared to only 10%. Figure 6 shows this effect by the resulting smaller cluster to the right. Due to the low specificity of CXR however, there is an increased risk of false positives associated with this approach.

**Figure 7:**
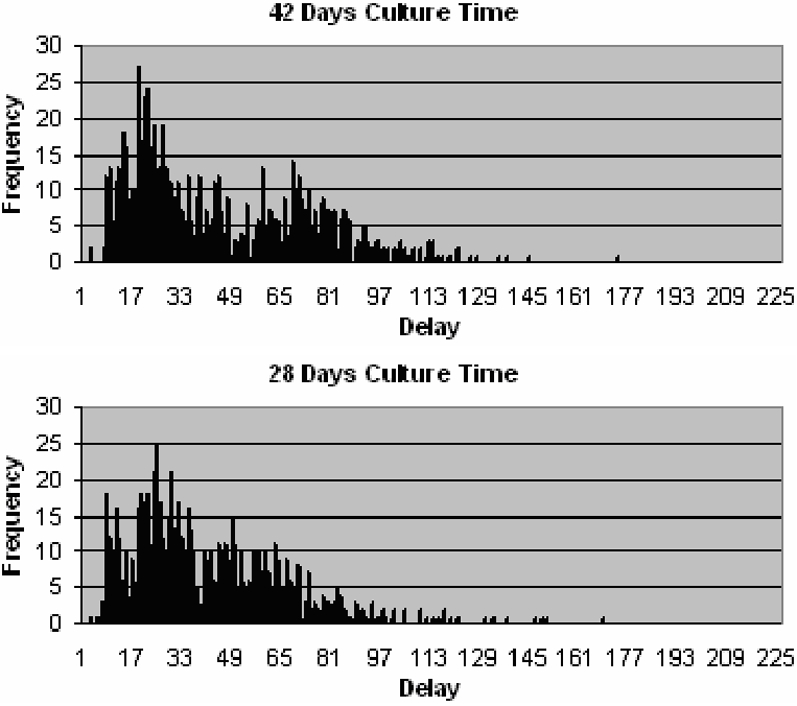
Distribution of delays since onset of symptoms to start of treatment for patients that do not drop out, when culture takes 42 days (top) and 28 days (bottom).

An alternative way of reducing delays associated with smear negative patients is to reduce culture time. More rapid culture methods are currently available, however they have other limitations. Figure 7 shows a shift to the left of the smear negative cluster when culture time is reduced to 28 days as is achievable using the MGIT method. LJ culture is currently the standard method and has a culture time of approximately 6 weeks.

**Figure 8:**
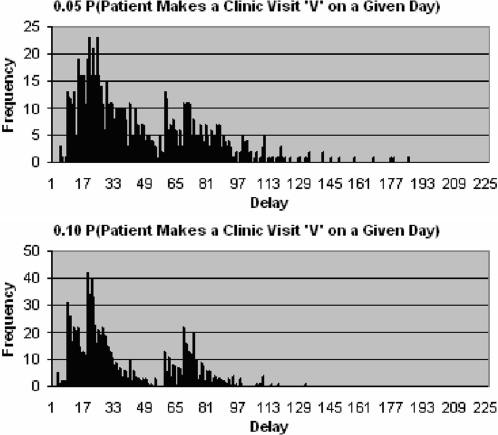
Distribution of delays since onset of symptoms to start of treatment for patients that do not drop out, with a 0.05 (top) and 0.10 (bottom) probability that patient makes a clinic visit on a given day.

Whether a TB patient is smear positive or negative, in order to reduce the overall delay it is clear that patients must seek the appropriate health provider as soon as possible after onset of symptoms. Figure 8 shows the effect of doubling the probability that a patient makes a clinic visit on a given day. When the probability is increased, mean delay is reduced, as diagnosis and thus treatment is started sooner. Specifically the distribution of delays has a sharper decline, as more patients experience the minimum delay. The four major distinguishable clusters seen in the lower histogram when viewed from left to right represent patients that are diagnosed mostly by the initial SSM test on their first visit, mostly by the initial SSM test on their second visit, mostly by the culture test on their first visit, and mostly by the culture test on their second visit. Reference to diagnosis made on a particular visit implies that patients make a visit to the TB test centre and a series of tests is initiated on that visit, but completed over a number of days.

**Figure 9:**
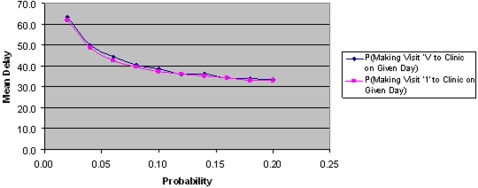
The effect of changing the probabilities of a patient making visit ‘1’ and ‘V’ on mean delay.

Figure 9 shows that delay is equally affected when only the probability of making the first visit is changed. This implies that the second and third visits are significantly less important in terms of overall delay.

**Figure 10:**
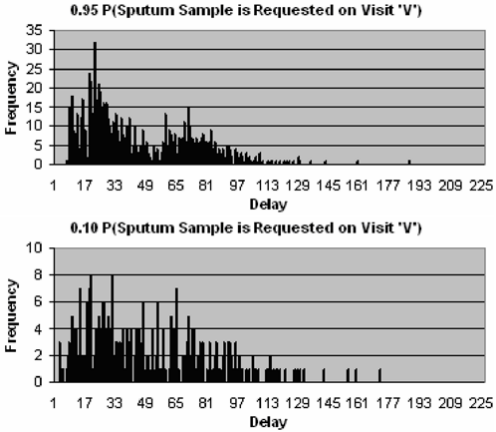
Distribution of delays since onset of symptoms to start of treatment for patients that do not drop out, with a 0.95 (above) and 0.10 (below) probability that sputum is requested on visit ‘V’.

**Figure 11:**
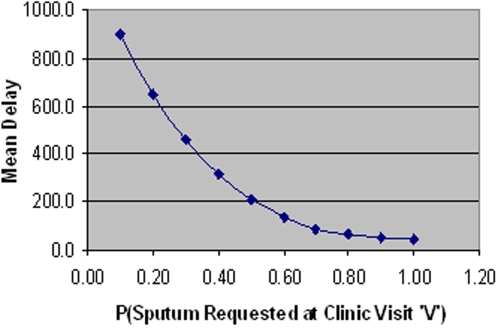
Relationship between the probability of sputum being requested on visit ‘V’ and mean delay.

Once a patient seeks the appropriate healthcare provider it is then important that the correct tests are carried out. Specifically a sputum sample should be requested if the healthcare provider suspects TB. Figure 10 shows that for those patients that do not drop out, the distribution of delays is significantly altered by a decrease in probability of sputum being requested. As the probability that sputum is requested on any of the visits decreases, the likelihood that a patient never receives a diagnosis is increased. Patients that do not receive a diagnosis remain untreated and delay is therefore equal to survival time. This explains why delay increases so rapidly when the probability of sputum being requested is low (figure 11).

**Figure 12:**
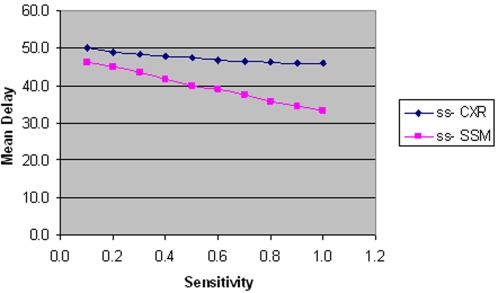
The effect of ss-CXR and SSM sensitivity on mean delay.

With regards to the sensitivity of smear negative tests, SSM and CXR both have a linear effect on mean delay. However this effect is significantly less than that of the initial SSM test due to smaller numbers of smear negative patients. The reason for the modest gains achieved from improving CXR sensitivity, as shown in figure 12, is that a positive (abnormal) result usually requires confirmation by further tests.

**Figure 13:**
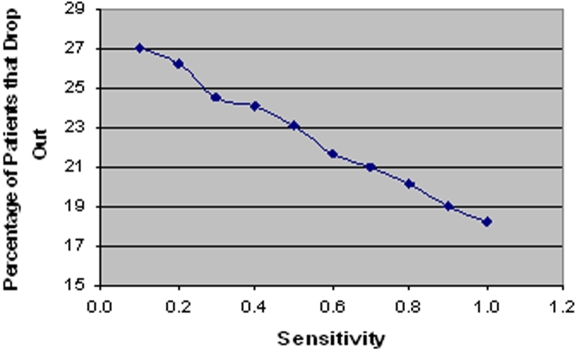
Linear relationship between initial SSM test sensitivity and percentage of patients that drop out.

A linear relationship is also found between initial SSM test sensitivity and the percentage of patients that drop out, as shown in figure 13. At each stage in the model a patient has the option of discontinuing diagnosis. Therefore, early diagnosis implies that fewer visits to the clinic will be required and patients will be less likely to drop out. A mean drop-out rate of 182 (out of 1000 cases) was recorded when test sensitivity is set to 1.0 and 270 when set to 0.1.

**Figure 14:**
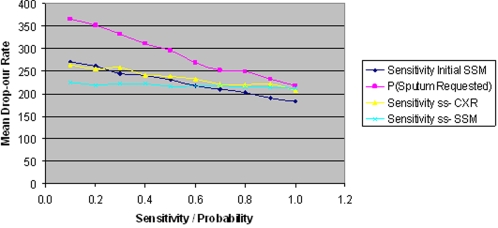
The effect of parameter changes on mean drop-out rate.

Figure 14 shows a comparison of the effects of varying different parameters on drop-out rate. Considering mean drop-out rate is 224 with standard parameter settings, this chart indicates that only by increasing initial SSM test sensitivity can sizeable reductions in the mean drop-out rate be achieved. Theoretically, if sensitivity was increased to 0.8, mean drop-out rate would be reduced to 202 and to 182 if sensitivity could be increased to 1.0.

Considering these parameter changes in isolation gives some insight into their individual effect on delay and drop-outs. A comparison of costs incurred by the healthcare system is also included. Table 3 shows a comparison of the potential benefits of such changes, relating them to different diagnostic scenarios.

**Table 3:** A comparison of different theoretical diagnostic scenarios.

Diagnostic method	Mean Delay	Mean Cost	Mean <1?show=[sr]?>Drop-out Rate
2 smears (Standard method)^1^	46.1	25.54	224.8
Same-day Diagnosis^2^	38.6		221.3
2 smears, treat ss-based on CXR^3^	38.7	21.50	218.7
2 smears, increase P(make visit)^4^	38.8	25.71	156.6
1-stop diagnosis^5^	43.4		219.8
2 smears^6^	43.8	24.10	219.6
3 smears^7^	45.2	28.00	219.6
2 smears HIV+patient cohort^8^	61.4	32.44	243.2
LJ culture^9^	66.3		183.2

**Notes:**

1. Sensitivity = 0.55, 2 days delay in producing SSM test results.

2. Sensitivity = 0.59 (same as for 3 smears), zero delay in delivering sputum, producing results and in patient collecting results.

3. Sensitivity = 0.55, 2 days delay in producing SSM test results, P(treating ss-based on CXR) = 0.5 (up from 0.1).

4. Sensitivity = 0.55, 2 days delay in producing SSM test results, P(make 1^st^ visit to clinic on given day) = 0.1 (up from 0.05).

5. Sensitivity = 0.59 (same as for 3 smears), zero delay in delivering sputum.

6. Sensitivity = 0.59 (same as for 3 smears due to reduced work load), 2 days delay in producing SSM test results.

7. Sensitivity = 0.59, 3 days delay in producing SSM test results.

8. Sensitivity = 0.36, rates of abnormal CXR kept the same, culture time for ss-patients = 8 weeks (up from 6), sensitivity ss-SSM kept the same.

9. LJ culture used in place of initial SSM test, culture time = 42 days, sensitivity = 1.0

The diagnostic approach used by the South African NTCP, which is based on using two smears in the initial SSM test, provides a benchmark in performance.

The following scenarios, listed in order of delay times from shortest to longest, were compared:

- A theoretical scenario using a test that achieves around 60% sensitivity and which can be processed in a single day while the patient waits.
- The proportion of ss-patients treated presumptively based on abnormal CXR results, increased from 0.1 to 0.5.
- The probability of making the first clinic visit, doubled to 0.1 on a given day.
- A so-called one-stop diagnosis using a test with 60% sensitivity.
- Diagnosis based on the use of two sputum smears.
- Diagnosis based on the use of three sputum smears.
- An HIV+ cohort of patients diagnosed by examination of two sputum smears. (Model parameters were altered, specifically sensitivity of the SSM test is lower and culture time is longer).
- Initial SSM is replaced by LJ culture.

A macro was used to increase each input parameter sequentially by 20% in order to determine the sensitivity of the model to each parameter (Table 4). From this analysis it is evident that increasing the sensitivity of the initial diagnostic test has a relatively large effect on mean delay, mean cost per patient and mean drop-out rate. Mean delay and cost per patient is decreased the most by this parameter change, causing around an 8% and 14% decrease respectively.

**Table 4:** Percentage Change in Mean Delay Due to a 20% Increase in Individual Parameters.

Parameters	Input Values	Average Mean Delay	% Change in Average Mean Delay
PPV	0.55	42.1	−8.07
NCHP	0.5	47.2	3.26
DNCHP	10	46.7	1.99
PHC1	0.05	43.8	−4.35
PHC2	0.05	46.0	0.50
PHC3	0.05	46.1	0.76
PD10	0.01	47.3	3.33
PD20	0.01	46.1	0.85
PD30	0.01	45.6	−0.32
PD11	0.01	45.8	0.06
PD21	0.01	46.4	1.46
PD31	0.01	46.7	2.04
PCXR1	0.10	47.0	2.84
PCXR2	0.10	46.1	0.79
PCXR3	0.10	45.8	0.01
SENCXR1	0.50	45.4	−0.69
SENCXR2	0.50	46.4	1.34
SENCXR3	0.50	46.7	2.14
DELCXR	5	47.2	3.13
PS1	0.95 *	45.2	−1.12
PS2	0.95 *	45.7	−0.18
PS3	0.95 *	44.9	−1.83
PD12	0.01	46.4	1.50
PD22	0.01	46.7	2.04
PD32	0.01	47.2	3.19
PD13	0.01	46.1	0.84
PD23	0.01	45.8	0.03
PD33	0.01	45.8	0.16
DEL11	1	46.3	1.22
DEL21	1	46.7	2.00
DEL31	1	47.2	3.26
DEL12	2	46.2	0.90
DEL22	2	45.7	−0.03
DEL32	2	45.9	0.32
DEL13	2	46.3	1.15
DEL23	2	46.7	1.99
DEL33	2	47.2	3.13
DELTR	2	46.3	1.30

**Note:** The average mean delay corresponding to a 20% increase in the particular parameter is compared to the standard mean delay of 45.7

### Conclusion

This modelling investigation highlights the numerous patient and healthcare system-related factors that contribute to the excessive total delay currently experienced by TB patients in many developing countries, before diagnosis and treatment is achieved. Such lengthy delays are, in-part, accountable for the inability to control the epidemic, as they allow time for a greater number of transmission events and subsequently for the disease to spread.[9]

The most important of the delay factors is the sensitivity of the initial SSM test. In developing countries, SSM is currently the most effective tool for diagnosing pulmonary TB, as it is rapid, cheap and has a high specificity; however it suffers from low sensitivity.[10] Often in practice, the sensitivity of Ziehl-Neelsen (ZN) smear is less than 50% when compared to culture.[11] TB patients that are not identified by this initial test incur relatively long delays as they must make additional hospital/clinic visits and undergo further testing. This study suggests that for every 0.1 increase in test sensitivity, a 3–5 day reduction in diagnostic delay is possible. Therefore in order to significantly reduce the mean total delay experienced by patients, the effectiveness of the initial test is crucial.

Our analysis draw attention to the excessive delays associated with smear negative TB patients, a finding that is supported in the literature.[12] This patient group experiences particularly long delays since they predominantly rely on sputum culture in order to obtain a decisive diagnosis. The conventional culture method using Lowenstein-Jensen (LJ) medium remains the so-called gold standard, and is still widely used, both for diagnosis of smear negative cases and drug sensitivity testing; however it requires up to 8 weeks to complete.[8], [13] Smear negative individuals are typically less infectious when compared to smear positive patients (approximately 20% relative infectiousness)[14] However due to the low sensitivity of SSM, the number of patients missed by the test makes up approximately half of all pulmonary TB cases and thus represent a significant proportion (∼10%, as calculated from the above fractions) of potential transmission events. The importance of introducing an appropriate new test for diagnosing smear negative cases in a timelier manner should therefore be emphasised.

The results from this investigation reinforce the importance that patients should promptly seek the appropriate medical attention following onset of TB symptoms. Mean delay is reduced by 7.3 days when the probability of a patient making the first visit to a clinic on a given day is doubled from 0.05 to 0.1. In order for this goal to be realised, the many obstacles that isolate different population groups from the appropriate care need to be overcome.[4], [15] Case finding is identified in the model as being a crucial factor with regards to diagnostic delay. Currently, a passive approach is used in many developing countries, however it may be necessary to adopt a more active approach if control of the epidemic is to be realised.

The probability that a sputum sample is requested emerged as an important issue, not surprisingly. For the standard scenario, the input value for this probability was high (0.95) and therefore increases in this parameter produce limited reductions in delay. However it is crucial that sputum is requested whenever there are indications of TB. Therefore healthcare workers need to be alert to the signs of TB particularly in lower risk groups such as children.

Simulations of two versus three smears used in the initial SSM test are compared in this study. It is clear that overall test sensitivity achieved by each approach is the crucial factor. While disparity between the use of two and three smears with respect to mean delay is small, the potential implications for the healthcare system are significant. Overburdening of healthcare systems has a particularly detrimental effect on TB diagnosis and where this is the case it has been suggested that the two smear approach should be considered.[16], [17]

The importance of factors that influence patient drop-out rates is also stressed; as such individuals will remain untreated and infectious in the community, resulting in potential TB transmission to their close contacts, including those caring for the sick person at the later stages of the disease.

With the ever increasing threat of MDR and XDR-TB as well as HIV, the goal posts for diagnosis have been moved. Continued development of diagnostic strategies is needed in order to maintain relevance. TB control is complex and it is clear that not all the criteria for case detection will be met by a single diagnostic method. How different methods are used concurrently is crucial to the success of control programs.
